# Cytokine Profiling in Immigrants with Clinical Malaria after Extended Periods of Interrupted Exposure to *Plasmodium falciparum*

**DOI:** 10.1371/journal.pone.0073360

**Published:** 2013-08-14

**Authors:** Gemma Moncunill, Alfredo Mayor, Azucena Bardají, Laura Puyol, Augusto Nhabomba, Diana Barrios, Ruth Aguilar, María-Jesús Pinazo, Mercè Almirall, Cristina Soler, José Muñoz, Joaquim Gascón, Carlota Dobaño

**Affiliations:** 1 Barcelona Centre for International Health Research (CRESIB, Hospital Clínic-Universitat de Barcelona), Barcelona, Catalonia, Spain; 2 Centro de Investigação em Saúde de Manhiça, Maputo, Mozambique; 3 Hospital Arnau de Vilanova, Lleida, Catalonia, Spain; 4 Hospital Santa Caterina de Salt, Girona, Catalonia, Spain; Burnet Institute, Australia

## Abstract

Immunity to malaria is believed to wane with time in the absence of exposure to *Plasmodium falciparum* infection, but immunoepidemiological data on longevity of immunity remain controversial. We quantified serum cytokines and chemokines by suspension array technology as potential biomarkers for durability of immunity in immigrants with clinical malaria after years without parasite exposure. These were compared to serum/plasma profiles in naïve adults (travelers) and semi-immune adults under continuous exposure, with malaria, along with immigrant and traveler patients without malaria. Immigrants had higher levels of IL-2, IL-5 and IL-8 compared to semi-immune adults with malaria (*P*≤0.0200). Time since immigration correlated with increased IL-2 (rho=0.2738*P*=0.0495) and IFN-γ (rho=0.3044*P*=0.0282). However, immigrants did not show as high IFN-γ concentrations as travelers during a first malaria episode (*P*<0.0001). Immigrants and travelers with malaria had higher levels of IFN-γ, IL-6, and IL-10 (*P*<0.0100) than patients with other diseases, and IL-8 and IL-1β were elevated in immigrants with malaria (*P*<0.0500). Therefore, malaria patients had a characteristic strong pro-inflammatory/Th1 signature. Upon loss of exposure, control of pro-inflammatory responses and tolerance to *P. falciparum* appeared to be reduced. Understanding the mechanisms to maintain non-pathogenic effector responses is important to develop new malaria control strategies.

## Introduction

*Plasmodium falciparum* infection still causes millions of malaria cases and deaths worldwide, mainly in sub-Saharan Africa [1]. The complex nature of the parasite and the lack of immune correlates of protection are impairing the development of a vaccine against malaria. In addition, the understanding of the mechanisms of induction and maintenance of immunological memory is very limited. Epidemiological data show that age and repetitive *P. falciparum* infections are key factors in naturally acquired immunity to malaria. Immunity to severe clinical symptoms and later to clinical malaria is achieved quite rapidly after few infections. However, immunity to parasitemia develops only after repeated infections over a number of years, it is not sterile and thus asymptomatic infections may exist throughout life [2].

Mechanisms of immunity to malaria are complex and include antibody and cellular responses that are required for both anti-parasitic and clinical immunity [3,4]. Cellular immune responses involved in immunity include (i) interferon (IFN)-γ and tumor necrosis factor (TNF) producing CD8^+^ T cells that inhibit parasite development and destroy infected hepatocytes, (ii) IFN-γ and memory CD4^+^ T cells that activate macrophages to phagocyte parasitized erythrocytes and merozoites, and (iii) regulatory T cells that control pathogenesis [4]. Despite the identification of these responses and several antigens putatively involved in protection, there is no biomarker that has reliably been shown to correlate with immunity. However, cytokines could be considered biomarkers of immunity and/or disease progression due to their prognostic role [5–7]. Cytokines and chemokines mediate cellular immune responses and they are responsible for the symptoms and pathological alterations during malaria disease. In fact, the outcome of the infection depends on the regulation of pro-inflammatory and anti-inflammatory immune responses, leading to protection or immunopathology [8].

It is commonly believed that anti-malarial immunity is short-lived and that continuous exposure to parasite antigens is needed to maintain it. In this line, it has been observed that severe disease and pro-inflammatory responses might not be less common among immigrants than among individuals who have not been previously exposed to malaria [9]. However, most clinical evidence indicate that after several years without exposure to *P. falciparum* infection, immigrants still maintain some immunity to clinical malaria, and their disease episodes are characteristically milder compared to naïve travelers with malaria [10–16]. Importantly, malaria epidemiology studies in areas of low and unstable transmission, such as South Africa and Madagascar, have shown that prior exposure, even several decades before, had a significant protective effect much later in life [17–19], suggesting persistence of immunological memory in the absence of re-infection. Therefore, it seems likely that people exposed to malaria do accumulate cellular immune memory, but few studies have investigated *Plasmodium* -specific cellular memory immune responses in malaria-exposed people. Remarkably, it was recently shown that antigen-specific IFN-γ and IL-2 T cell responses, as well as γδ T cells, can remain undiminished up to 14 months after a single *P. falciparum* experimental infection [20]. Under natural exposure conditions, IFN-γ CD4^+^ T cell responses to *P. falciparum* appeared to be short-lived (half-life of 3.3 years) in areas of unstable malaria transmission, whereas IL-10 CD4+ T cells did not appear to decline for 6 years [21]. In another study, regulatory T cells circulating during acute malaria episode almost exclusively expressed an activated memory phenotype suggesting that they expanded from a pre-existing pool of memory T-cells [22].

In this study, we aimed to identify peripheral cytokines and chemokines during a malaria episode as potential biomarkers for maintenance or loss of immunity after an extended cessation of exposure to *P. falciparum*. We recruited African immigrants living in Spain for an average 7 years returning from a malaria endemic area with a malaria episode. Cytokine and chemokine serum levels on admission were compared with those of naïve travelers with a first clinical malaria episode, and semi-immune adults from a malaria endemic area of Mozambique presenting to hospital with clinical malaria. Results provide insights into immune responses that might be key for the induction and maintenance of immunity to clinical malaria in relation to history of exposure to *P. falciparum* and could help in the identification of cytokine/chemokine prognosis markers.

## Methods

### Ethics Statement

Written informed consent was obtained from participants before sample collection. Approval for the protocols was obtained from the Hospital Clínic of Barcelona Ethics Review Committee and the National Mozambican Ethics Review Committee. Parasitemic individuals were treated according to standard national guidelines at the time of the studies. The antimalarial drug regimen used to treat patients in Spain was Malarone (atovaquone/proguanil) or quinine plus doxycycline if intravenous treatment was needed and in Mozambique the treatment was artesunate plus sulphadoxine-pyrimethamine.

### Study design, subjects and sample collection

Patients attending the Tropical Medicine Units at Hospital Clínic de Barcelona (Barcelona, Spain), Hospital Arnau de Vilanova (Lleida, Spain) and Hospital Santa Caterina de Salt (Girona, Spain) between 2005 and 2009 were invited to participate. Sick volunteers enrolled in the study were African adults residing in Spain (immigrants, n=55) and adults from non-African origin without previous episodes of malaria (travelers, n=22) [23] who had been diagnosed with *P. falciparum* malaria after traveling to an African country. Malaria was defined by the presence of *P. falciparum* on Giemsa-stained blood smears detected by light microscopy together with fever and other clinical signs of malaria. Parasitemia in blood was assessed by thin blood smears by examining 10 to 100 high power fields and counting from 1,000 erythrocytes up to 10,000 erythrocytes depending on the parasite numbers, and expressed as the percentage of parasitized erythrocytes. In addition, 38 immigrants or travelers attending the Tropical Medicine Units presenting with other diseases but without malaria were also recruited (Table 1). Most of them had a febrile syndrome or traveler diarrhea, but also giardiasis, katayama syndrome, mononucleosis syndrome EBV, pneumonia, pruritus eczema, anxiety disorder, appendicitis, dermatitis, toxic syndrome, viral infection, ketoacidosis, diabetes, headache, spontaneous abortion, bacterial lung abscess, HIV infection were diagnosed. Blood samples from acute malaria episodes (day 0) and at convalescence after malaria treatment (days 7 and 28) and blood samples from non-malaria patients were collected by venipuncture into one vacutainer without anticoagulant for serum cryopreservation at -80^°^ C. Clinical and demographical data were recorded in standardized questionnaires. Data on cytokine levels in serum from travelers have been previously published [23], but are re-analysed here for comparison to the immigrant group.

**Table 1:** Description of study participants.

	**Immigrants**		**Travelers**		**Semi-Immunes**
**Characteristics**	**Malaria**	**No Malaria**	**Malaria**	**No malaria**	**Malaria**
N					
Day 0	55	17	22	21	90
Day 7	31	na	14	na	na
Day 28	11	na	6	na	na
Age, median IQR (years)^d^	34 (29,43)	36 (30,44)	31 (28,38)	31 (28,38)	26 (19,36)
Sex^e^, n (%)					
Males	40 (72.7)	7 (41.18)	15 (75)	9 (42.86)	52 (53)
Origin area, n (%)					
Europe	0 (0)	0 (0)	17 (85)	21 (100)	0 (0)
Africa	54 (100)	17 (100)	0 (0)	0 (0)	90 (100)
Others	0 (0)	0 (0)	3 (15)	0 (0)	0 (0)
Time since immigration, median IQR (years)	7 (5,14)	4 (1,8)	na	na	na
Number of returns, n (%)					
0	5 (9.6)	6 (42.86)	na	na	na
1-2	11 (21.2)	2 (14.29)	na	na	na
3-4	26 (50)	3 (21.43)	na	na	na
>5	10 (19.2)	3 (21.43)	na	na	na
Parasitemia by microscopy					
median IQR (%)^f^	0.4 (0.02; 1.5)	na	0.075 (0.01;0.8)	na	nd
median IQR (parasites/μl)	nd	na	nd	na	35379 (14338; 61176)
Symptoms, n (%)					
Fever	na	7 (41.18)	na	12 (57.14)	na
Nauseas, epigastralgia	na	3 (17.65)	na	2 (9.52)	na
Discomfort, arthralgia, anxiety	na	3 (17.65)	na	1 (4.76)	na
Respiratory infection	na	1 (5.88)	na	1 (4.76)	na
Cough	na	1 (5.88)	na	0 (0)	na
Ketoacidosis	na	1 (5.88)	na	0 (0)	na
Lung abscess	na	1 (5.88)	na	0 (0)	na
Diarrhea n (%)	na	0 (0)	na	4 (19.05)	na
Skin lesion	na	0 (0)	na	1 (4.76)	na

Abbreviations: na, not applicable; nd, not determined; IQR, Interquartile range

^a^ Immigrants returned from visiting their countries of origin: Cameroon (n=3, 5.5%), Ghana (n=8, 14.6%), Guinea-Conakry (n=4, 7.3%), Equatorial Guinea (n=12, 21.8%), Gambia (n=8, 4.6%), Mali (n= 4, 7.3%), Mauritania (n=1, 1.8%), Mozambique (n=1, 1.8%), Nigeria (n=6, 10.9%) and Senegal (n=7, 12.7%). Data was missing for one immigrant.

^b^ Immigrants without malaria were from Benin (n=1, 5.9%), Burkina Faso (n=2, 11.8%), Guinea-Conakry (n=2, 11.8%), Equatorial Guinea (n=2, 11.8%), Gambia (n=1, 5.9%), Kenya (n=1, 5.9%), Mali (n=3, 17.7%), Mauritania (n=1, 5.9%), Mozambique (n=1, 5.9%), Nigeria (n=1, 5.9%), Senegal (n=1, 5.9%) and Sudan (n=1, 5.9%).

^c^ Travelers came from Burkina Faso & Mali & Senegal (n=1, 5.0%), Burkina Faso (n=3, 15.0%), Burkina Faso & Mali & Ghana & Togo (n=1, 5.0%), Ivory Coast (n=1, 5.0%), Guinea-Conakry (n=1, 5.0%), Equatorial Guinea (n=3, 15.0%), Gambia & Senegal (n=1, 5.0%), Madagascar (n=1, 5.0%), Mali (n=1, 5.0%), Mozambique (n=2, 10.0%), Mozambique & South Africa (n=1, 5.0%), Senegal (n=3, 15.0%) and Sierra Leone & Senegal (n=1, 5.0%). Data was missing for two travelers.

^d^ *P*=0.0001 Kruskal Wallis test.

^e^ *P*=0.0140 χ^2^ test.

^f^ *P*=0.0890 Mann-Whitney test.

Additionally, 90 semi-immune adults with life-long exposure to *P. falciparum* were recruited in the context of a hospital-based study conducted at the Centro de Investigaçao em Saúde de Manhiça (Manhiça, Mozambique), where malaria transmission is perennial, with some seasonality and of moderate intensity. Non-pregnant women and men patients attending the Manhiça District Hospital with a diagnosis of *P. falciparum* clinical malaria in 2006 were enrolled into the study [24]. Clinical malaria was defined as the presence of asexual *P. falciparum* parasites on blood smears, together with fever. Blood slides were read to quantify parasitemia following standard quality-controlled procedures at the CISM laboratory. Blood films were Giemsa-stained, and examined using a light microscope. Parasite density was assessed by counting the number of asexual stage parasites until 500 leukocytes or parasites had been counted. Slides were declared negative only after 2,000 leukocytes had been counted. Parasite numbers were converted to a count/mL by assuming a standard leukocyte count of 8,000/mL. All sides were read by two independent microscopists and a third reading was performed if there was discrepancy in positivity or the ratio of densities from the two readings was more than 1.5 or the absolute difference was 10 parasites/mL. Blood samples were collected by venipuncture into heparinized vacutainers and plasma samples cryopreserved at -80^°^ C.

### Cytokine and chemokine levels

Concentrations (pg/mL) of interleukin (IL)-12p70, IL-2, IFN-γ, IL-4, IL-5, IL-10, IL-8, IL-6, IL-1β, TNF and TNF-β in plasma and serum were measured using a commercial multiplex suspension array kit (Human Th1/Th2 11plex FlowCytomix kit, Bender MedSystems, Austria) and flow cytometry. This kit was chosen after comparison of several commercial kits to measure cytokine responses to *P. falciparum* [25]. Twenty-five µL of plasma or serum were tested following manufacturer’s instructions and one positive control was used in each plate for qualitative evaluation of the assay performance. Mean fluorescence intensity (MFI) from microspheres was acquired with a BD FACSCanto II and analyzed in FlowCytomix Pro2.2.1 software (Bender MedSystems). A 7-point dilution standard curve supplied by the manufacturer was performed in duplicates in each assay, and concentration of each analyte was obtained by interpolating MFI to a 5-parameter logistic regression curve automatically calculated by the FlowCytomix software for each analyte. Any value below the limits of detection was given a value of half the detection limit for that cytokine or chemokine.

### Statistical methods

Categorical variables were presented as frequencies or percentages, and their comparison between patient groups was done using chi-square test or Fisher’s exact test. For non-normally distributed continuous variables, medians and interquartile ranges (IRQ) were shown and their comparison between groups was done using the non-parametric Kruskal Wallis test or the Mann–Whitney U test. TNF-β was excluded from the statistical analysis since concentration in most samples was below the limit of detection (14/267 [5.24%]) as we have observed before in previous studies [8,23]. Correlations within groups were assessed by Spearman’s rank coefficient. *P*-values <0.05 were considered statistically significant. Although Bonferoni tests were performed, crude p values reported in this exploratory study were not adjusted for multiple comparisons and were interpreted for internal coherence, consistency of results and biological plausibility. All data collected were analyzed using Stata version 11.0 (Stata Corporation, College Station, TX, USA).

## Results

### Description of participants

Table 1 shows the characteristics of the study participants. Age was lower in semi-immune adults compared to immigrants and travelers (*P*>0.0001) and there were more males in the immigrant and traveler groups with malaria than in the other groups (*P*=0.0140). Immigrants were original of different African countries and most of the travelers were from Europe. Visiting countries were very heterogeneous among immigrants and travelers. Immigrants returned from visiting their countries of origin. Immigrants with malaria had lived for a median of 7 years in Spain and 9.6% had never returned to their original country before, 21.1% had returned 1 to 2 times, 50% 3 to 4 times and 19.2% had returned more than 5 times. Immigrants without malaria had a different time since immigration, but this difference was not statistically significant. No significant differences were detected in parasitemias between immigrants and travelers (Table 1).

### Differential cytokine profiling in immigrants compared to semi-immune adults

**Figure 1:**
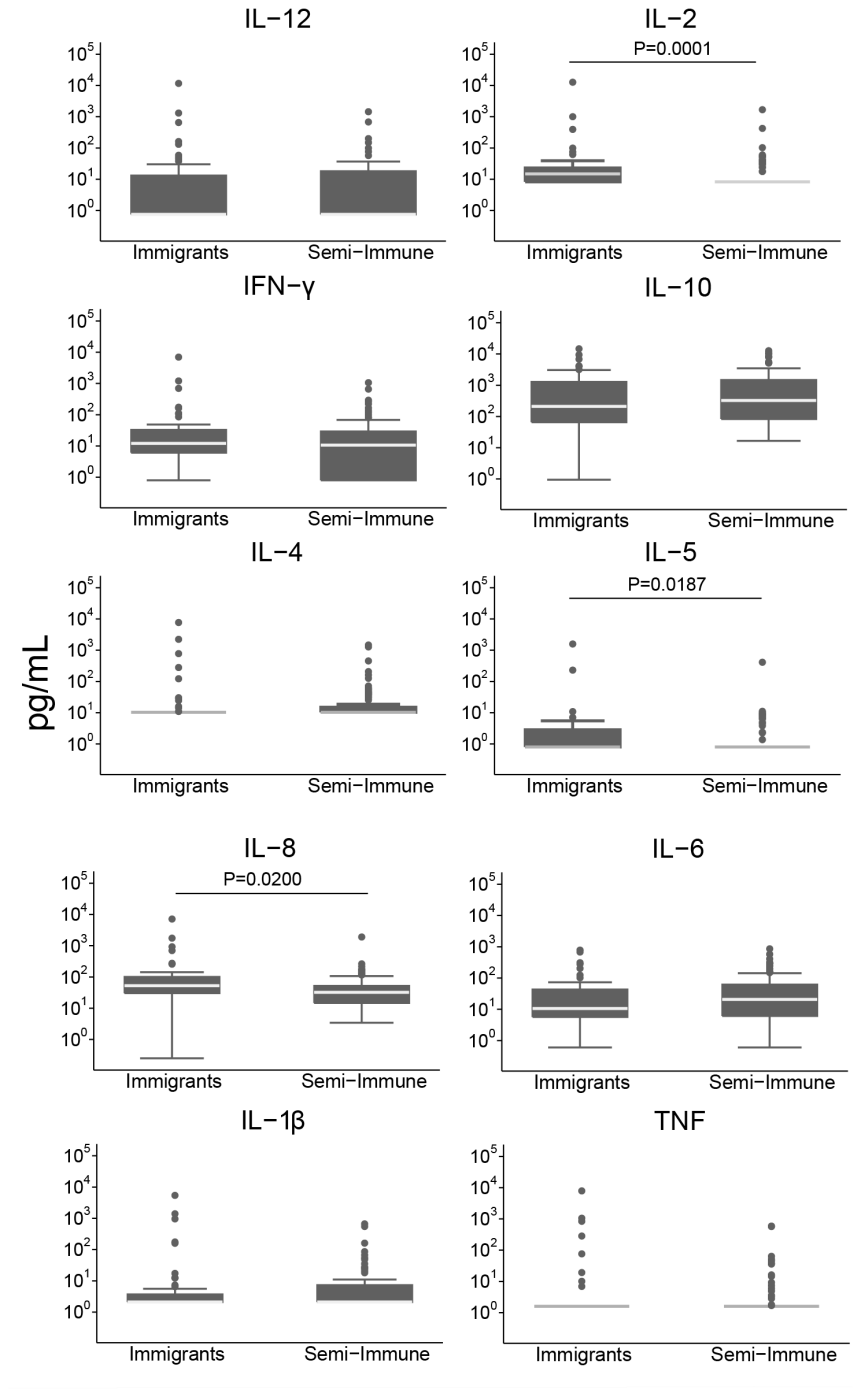
Effect of loss of exposure on cytokine responses in immigrants compared to semi-immune individuals, all with a malaria acute episode. Cytokines and chemokines were measured in serum from immigrants and plasma from semi-immune adults by a multiplex suspension array kit and flow cytometry. The boxplots illustrate the medians and the 25^th^ and 75^th^ quartile and the whiskers represent the 10% and 90% percentiles. Outliers are marked with circles. A Mann Whitney U test was performed for each comparison, and significant *P* values (*P*<0.05) are shown.

Immigrants showed a different cytokine profile than semi-immune adults during an acute malaria episode (Figure 1). Immigrants had significantly higher serum IL-2 (median [IQR] of 14.74 [8.20; 22.97] pg/mL), IL-5 (0.80 [0.80; 2.87] pg/mL) and IL-8 (52.2 [32.72; 114.69]) pg/mL levels compared to plasma levels in semi-immune adults (8.20 [8.20; 8.20] pg/mL, *P*=0.0001; 0.80 [0.80; 0.80] pg/mL, *P*=0.0187; and 32.32 [14.69; 51.61] pg/mL, *P*=0.0200, respectively). However, only IL-2 differences remained statistically significant after applying a correction test for multiple comparisons.

**Figure 2:**
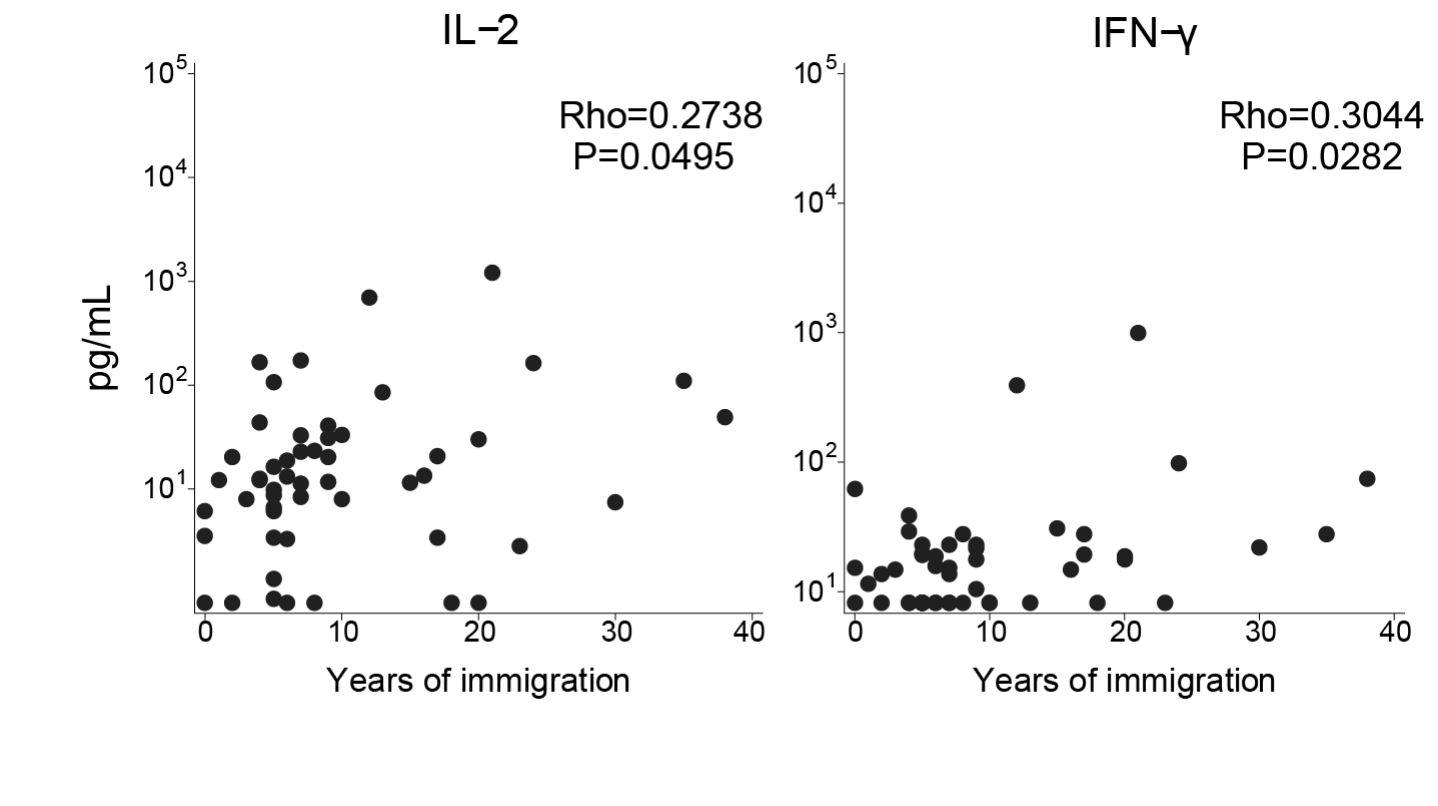
Effect of time of immigration on IL-2 and IFN-γ serum concentrations. Cytokine concentrations in serum from immigrants were measured by multiplex suspension array kit and flow cytometry and correlated with time of immigration. Only significant correlations are shown. Correlations were performed using Spearman’s test, n=52.

To determine the effect of time since immigration on the cytokine responses in a malaria acute episode, Spearman correlation coefficients were calculated for cytokines and years since immigration (Figure 2). IFN-γ and IL-2 correlated positively with time since immigration.

### Differential cytokine profiling in immigrants compared to naïve travelers

**Figure 3:**
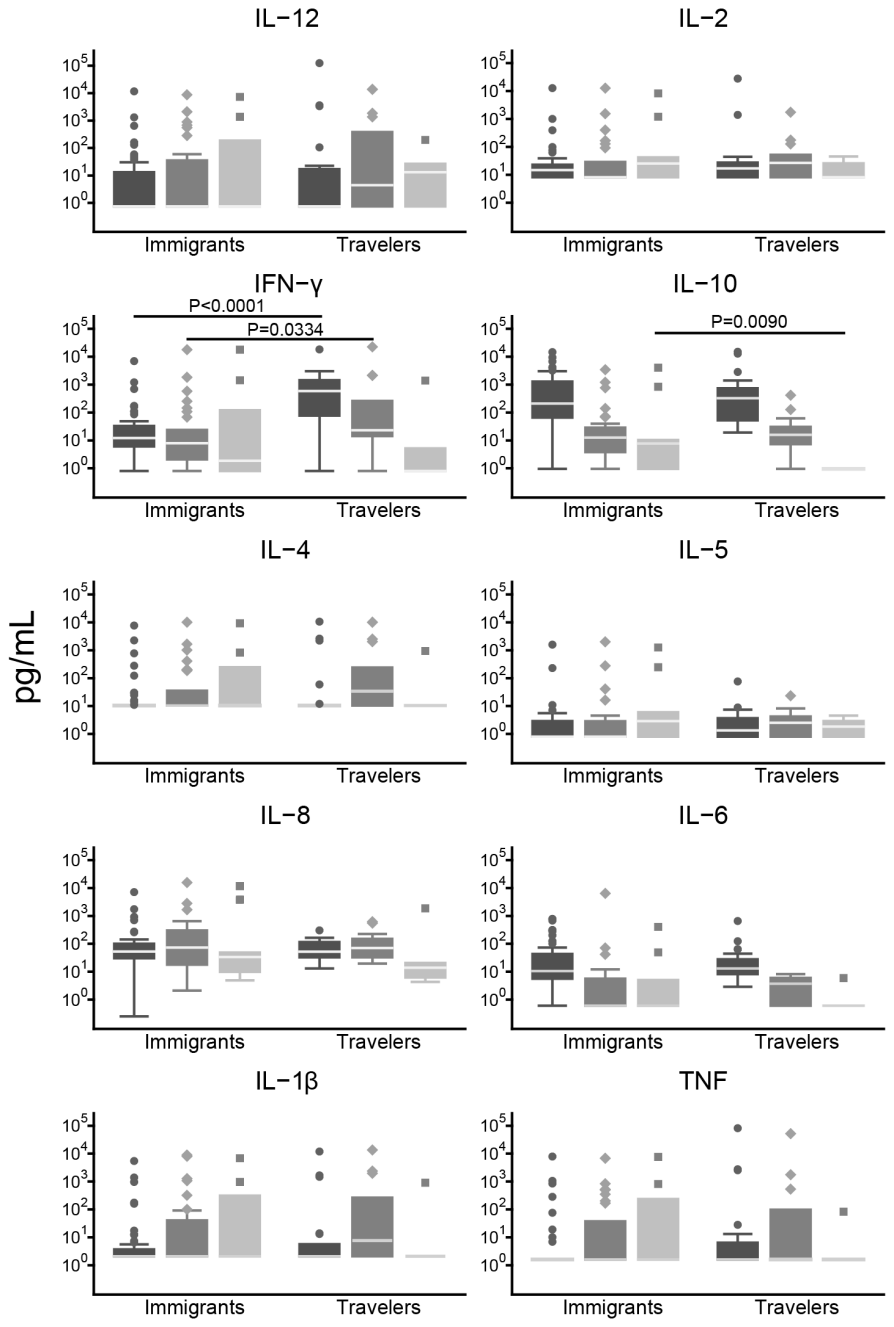
Effect of loss of exposure on cytokine responses in immigrants compared to naïve individuals, all with a malaria episode. Serum cytokine and chemokine concentrations in immigrants and travelers at different time-points during and after a malaria episode: during an acute malaria episode (day 0, black boxes) and at convalescence (day 7 dark grey, day 28 light grey). Data are presented as boxplots that illustrate the medians and the 25^th^ and 75^th^ quartile and the whiskers represent the 10% and 90% percentiles. Outliers are marked with circles. A Mann Whitney U test was performed for each comparison, and significant *P* values (*P*<0.05) are shown.

Cytokine and chemokine serum levels were measured in all immigrants and travelers during the acute malaria episode and in a subset of patients during convalescence (31 immigrants and 14 travelers at day 7; 11 immigrants and 6 travelers at day 28; Figure 3). Immigrants had lower concentrations of IFN-γ in an acute episode of malaria (median [IQR] of 12.1 [6.11; 32.88] pg/mL) and at day 7 of convalescence (7.97 [2.09: 23.78] pg/mL) compared to naïve adults with a first episode of malaria (584.535 [77.17; 1446.56] pg/mL, *P*<0.0001; and 23.23 [14.38; 259.39] pg/mL, respectively, *P*=0.0334). Immigrants also had higher levels of IL-10 at day 28 of convalescence (7.74 [0.95; 10.25] pg/mL) compared to naïve adults (0.95 [0.95: 0.95] pg/mL, *P*=0.0090). However, only differences in IFN-γ levels remained statistically significant after correcting for multiple comparisons.

To evaluate the magnitude of the cytokine/chemokine responses as well as its kinetics from acute to convalescent phases, ratios of cytokine/chemokine concentrations between days 0, 7 and 28 were calculated. Day 0 to day 7 ratios of IL-4 and IL-1β were significantly higher in immigrants compared to travelers (*P*=0.0023 and *P*=0.0084, respectively). In immigrants, there was also a trend to have higher ratio of IL-12 (*P*=0.0513) and TNF (*P*=0.0684), and lower ratio of IFN-γ (*P*=0.0954). There were no differences in day 0 to day 28 ratios (data not shown).

### Cytokine and chemokine responses correlating with parasite densities

IL-10 and IL-6 levels correlated positively with parasitemias in immigrants and travelers (Table 2). Levels of IL-8 only correlated with parasitemia in travelers, and IL-1β in immigrants. None of the cytokines/chemokine tested correlated with parasite density in semi-immune adults.

**Table 2:** Relevant Spearman correlations of parasitemia or parasite density with serum or plasma cytokine/chemokines.

	**Immigrants**		**Semi-Immunes**		**Travelers**	
Cytokines	rho	*P*	rho	*P*	rho	*P*
IL-10	0.4760	*0.0004*	0.1064	*0.3182*	0.6324	*0.0028*
IL-8	0.1876	*0.1875*	-0.0451	*0.6731*	0.5061	*0.0228*
IL-6	0.2772	*0.0489*	0.0740	*0.4880*	0.7277	*0.0003*
IL-1β	0.2819	*0.0451*	-0.0441	*0.6797*	0.4873	*0.4873*

Cytokine/chemokine concentrations did not show any correlation with age or any association with the area of birth or with the travel destination where immigrants and travelers became infected (data not shown). Also, there were no correlations among cytokines/chemokines and antibody responses to *P. falciparum* antigens [26] in these patients (data not shown).

### Cytokine and chemokine profiles during clinical malaria episodes

**Figure 4:**
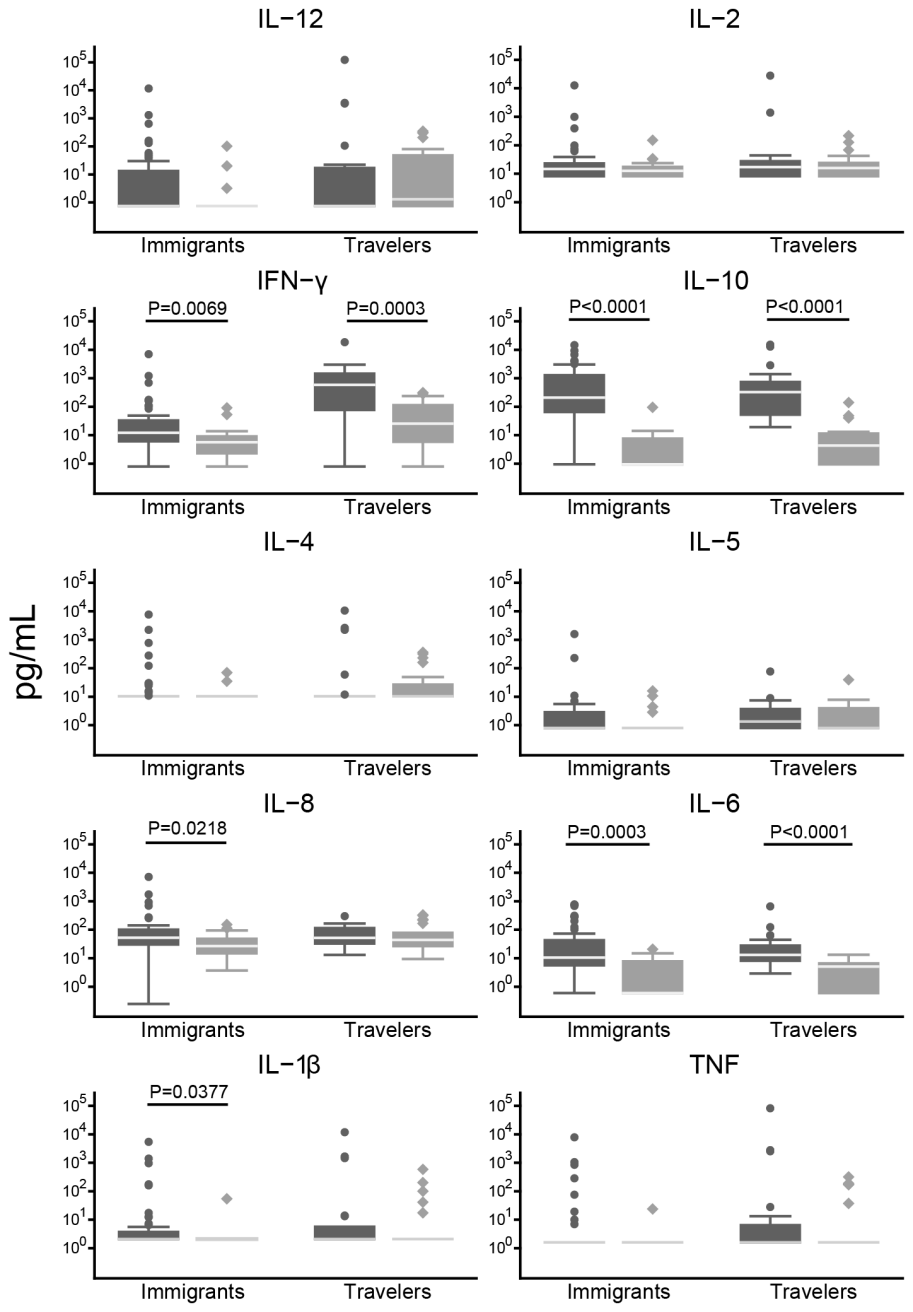
Serum cytokine and chemokine profiles in patients with malaria or with other diseases. Cytokines and chemokines were measured in serum of immigrants and travelers with malaria (dark grey boxes) and in serum of immigrants and travelers with other diseases (light grey boxes). Data are presented as boxplots that illustrate the medians and the 25^th^ and 75^th^ quartile. Whiskers represent the 10% and 90% percentiles and outliers are marked with circles. A Mann Whitney U test was performed to compare groups with malaria with groups with other diseases, and significant *P* values (*P*<0.05) are shown.

Patients presenting to the Tropical Medicine Units with an acute malaria episode showed a different cytokine/chemokine profile than patients presenting with other symptoms (Table 1). Travelers and immigrants with clinical malaria had an overall stronger cytokine/chemokine response (Figure 4). IFN-γ levels were higher in travelers (median [IQR] of 584.535 [77.17; 1446.56] pg/mL) and immigrants (12.1 [6.11; 32.88] pg/mL) with malaria, compared to individuals with other diseases (24.3 [5.88; 113.11] pg/mL, *P*=0.0003 and 5.64 [2.81; 8.43] pg/mL, *P*=0.0069, respectively). IL-6 levels were also higher in travelers (13.085 [8.17; 27.69] pg/mL) and immigrants (10.59 [5.65; 43.1] pg/mL) with malaria than in patients without malaria (5.26 [0.6; 6.61] pg/mL, *P*=0.0003 and 4.98 [0.6; 7.8] pg/mL, *P*<0.0001, respectively). Travelers and immigrants with malaria also had higher levels of IL-10 (325.68 [52.71; 740.19] pg/mL and 210.1 [65.55, 1277.3] pg/mL, respectively) compared to individuals with other diseases (7.7 [0.95, 11.47] pg/mL, 325.68 [52.71, 740.19] pg/mL, respectively, *P*<0.0001). IL-8 and IL-1β were higher only in immigrants with malaria (52.45 [30.33; 100.25] pg/mL and 2.1 [2.1; 3.7] pg/mL, respectively) compared to immigrants with other diseases (37.86 [14.81; 48.08] pg/mL, *P*=0.0218 and 2.1 [2.1; 2.1] pg/mL, *P*=0.0377, respectively).

## Discussion

Estimating duration of immune memory against clinical malaria in malaria-endemic populations is complicated due to re-exposure and boosting. Migrants moving from malaria endemic to non-endemic areas offer a good opportunity to study persistence of immunity and associated immune markers. This study was conducted in immigrants presenting with clinical malaria after returning from endemic areas, to assess how the loss of exposure affected their acute and convalescent peripheral blood cellular immune responses.

First, when cytokine/chemokine blood levels in immigrants were compared to those in semi-immune individuals, both with clinical malaria, a different profile was observed. Loss of exposure was associated with increased levels of IL-2, IFN-γ, IL-8 and IL-5. In particular, immigrants showed higher serum concentrations of IL-2, IL-8 and IL-5 in acute malaria compared to semi-immune adults, although only IL-2 and IFN-γ showed to be dependent on time since migration. It is possible that recent visits to endemic countries or the total number of returns (69% of immigrants had returned at least 3 times) may have represented some malaria re-exposure that could have diluted the effect of time since immigration. The fact that cytokines/chemokine were measured in serum in immigrants and in plasma in semi-immune adults may have introduced certain error as it has been described that measurements may differ, depending on the cytokine, if the matrix plasma or serum [27,28], but we do not think that it affected significantly our results.

Second, when compared with naïve adults presenting a first malaria episode, immigrants with malaria had lower levels of IFN-γ, suggesting that even if there was a decline in cellular immunity this loss was not complete. In this line, we had previously observed that this group of travelers had higher levels of IFN- γ compared to malaria-exposed individuals (after few or continuous exposure) [23]. Nevertheless, similar parasitemias were observed in immigrants and travelers suggesting that there are no differences in parasite-controlling immunity, but rather in the cellular responses controlling disease. This is consistent with our clinical observations that indicate a certain maintenance of protective immunity in immigrants, despite some loss of responses upon cessation of malaria exposure [14,15].

The cytokines/chemokine measured come from peripheral blood cells as well as from endothelial cells [29] and site specific responses may not be reflected. IFN-γ is produced by natural killer (NK) cells, γδ-T cells, CD8^+^ T cells and T_H_1 CD4^+^T cells. IL-2 is mainly produced by T cells and reflects a strong cellular activation and proliferation. IL-2 stimulates NK differentiation and proliferation as well as B cell production of immunoglobulins. IL-8 is an important chemokine mediator of inflammation produced mainly by macrophages and endothelial cells, which recruits innate cells such macrophages, granulocytes and stimulates phagocytosis. IL-5 is produced by T_H_2 CD4^+^ T cells, but also eosinophils and mast cells and is involved in eosinophil activation, B cell growth and immunoglobulin secretion. Therefore, all these cytokines/chemokine are probably (mostly) reflecting a strong innate immune activation, although this should be demonstrated with data of cytokine producing cells. Not all patients were febrile at the time of day 0 serum sampling, although most of the patients had a history of fever during the previous days. This is of importance as some cytokines such IL-6, TNF, and IL-1β, considered pyrogens [30,31], may fluctuate with fever, so we are probably detecting lower levels of these proinflammatory cytokines. This would explain why our patients had such low levels of IL-1β and TNF compared to other studies [32]. Nevertheless, immigrants and travelers with malaria had higher IFN-γ, IL-6 and IL-10 compared to those without malaria. Similar findings have been previously shown in other studies [32–34] and excessive production of pro-inflammatory cytokines such as IL-6 has been associated to severe malaria [8,33] as well as high levels of IL-10 [32,33], although specific patterns of cytokines have been found to vary depending on the different clinical presentations of severe malaria [32,33].

We wanted to explore if parasitemia or parasite density was directly affecting the production of cytokines. There were no significant differences in parasitemia between immigrants and travelers, but parasite density data could not be compared with those of semi-immune people due to different slide reading methods. The assessment of correlations between each of the cytokines/chemokine and parasitemia showed that IL-2 and IFN-γ levels were independent from parasitemias, suggesting that other mechanisms could play a role in regulating those T_H_1 cytokines. Furthermore, IL-10 and IL-6 positively correlated with parasitemia in immigrants and travelers, whereas IL-1β only correlated with parasitemia in immigrants, and IL-8 only in travelers. Of note, semi-immune individuals did not show any correlation between cytokine/chemokine concentrations and parasite density. This may reflect a better capacity to regulate or mitigate the immune pro-inflammatory response induced in an acute infection, probably through mechanisms of tolerance that could result in milder malaria [35].

Data about the persistence of protective immune responses is controversial and may depend on the antigen and the immune response assessed [20,21,36–38]. In our study, we found increased serum cytokines/chemokine associated with loss of exposure, reflecting a more prominent T_H_1 and pro-inflammatory cellular response, characteristic of non-immune patients with malaria compared with patients with other diseases. This is in line with what might be expected with increasing time since last malaria exposure: a shift in the cytokine/chemokine balance from an anti-inflammatory response towards a more pro-inflammatory response, reflecting a loss of malaria tolerance [35]. Thus, immune responses that limit appearance of clinical symptoms may be lost more easily (e.g. potentially related to rapid decay of antibodies against glycosylphophatidylinositol after leaving an endemic area [39,40]) than for example, immune responses controlling parasite density. This would explain that previously immune patients appear to make strong inflammatory responses to rather low numbers of parasites, feeling ill but recovering rapidly, and with lower risk of developing severe malaria or dying compared to travelers [10,11]. However, the elevated IFN-γ response in immigrants does not seem consistent with the reported protective role of this cytokine [41], and the relatively short half-life of IFN-γ effector CD4^+^ T cells described recently in an area of low transmission in Thailand [21]. Nevertheless, the Thai study measured CD4^+^ T cell memory responses, whereas the IFN-γ concentration in plasma may come from other cell types, reflecting a predominantly innate response rather than an acquired one.

A limitation of this study could be that immigrants and naïve adults were originally from very diverse countries (African or European), whereas semi-immune adults were from a unique African endemic area (Mozambique); thus, this could add genetic or environmental confounding factors. However, the immune response in clinical malaria is so pro-inflammatory compared to other diseases that it probably overcomes these limitations.

In summary, immigrants returning from endemic areas with malaria had higher serum concentrations for some cytokines/chemokines (IL-2, IL-5, IL-8) compared to semi-immune adults with malaria, suggesting that this profile is associated with a partial loss of immunity. Time since immigration, and therefore, loss of exposure, correlated with increased T_H_1 cytokines IL-2 and IFN-γ. However, immigrants did not show as high IFN- γ response as naïve adults in a first malaria episode, reflecting some persistence of responses associated with semi-immune individuals. Taken together, these observations may imply that immune mechanisms involved in malaria tolerance may be lost, explaining why previously immune patients appear to make strong inflammatory responses to clinical malaria. In addition data point out to immune responses that need further study to develop strategies to induce or potentiate immunity to clinical malaria.
